# The Predictive Properties of Psychiatric Diagnoses, Dynamic Risk and Dynamic Risk Change Assessed by the VRS-SO in Forensically Admitted and Released Sexual Offenders

**DOI:** 10.3389/fpsyt.2019.00922

**Published:** 2020-01-08

**Authors:** Reinhard Eher, Sandra Hofer, Anna Buchgeher, Stefan Domany, Daniel Turner, Mark E. Olver

**Affiliations:** ^1^ Federal Evaluation Centre for Violent and Sexual Offenders (FECVSO), Ministry of Constitutional Affairs, Reforms, Deregulation and Justice, Vienna, Austria; ^2^ Violence Research and Prevention Centre (IGF), Vienna, Austria; ^3^ Department of Psychiatry and Psychotherapy, University Medical Center Mainz, Mainz, Germany; ^4^ Department of Psychology, University of Saskatchewan, Saskatoon, SK, Canada

**Keywords:** sexual offenders, psychiatric placement, risk assessment, risk change, Violence Risk Scale–Sexual Offense Version (VRS-SO), psychiatric diagnoses

## Abstract

Psychiatric diagnoses, static risk factors, and criminogenic needs at time of admission and release were examined in a mentally ill sample of psychiatrically detained sexual offenders. Although clinically found to be at low or even very low risk at discharge, 12% reoffended sexually over an average follow-up of 7 years. Psychotic disorders were present in only 5% of offenders, whereas 93% had a personality disorder diagnosis and 76% a paraphilic disorder diagnosis. Only exhibitionism and alcohol misuse were associated with relapse. Static risk factors captured by the Static-99 also did not significantly predict recidivism; however, the VRS-SO—a structured risk assessment tool that assesses criminogenic needs and changes in risk from treatment or other change agents, rated retrospectively on the present sample—predicted sexual recidivism as well as any new imprisonment or psychiatric placement. In particular, the sexual deviance factor of the VRS-SO had large in magnitude predictive associations with sexual reoffending, while treatment related changes assessed on this factor were significantly related to non-reoffending. Findings corroborate the advantages of structured risk assessment and structured change monitoring, particularly for complex clientele such as mentally ill sexual offenders.

## Introduction

The rate of admission of sexual offenders to forensic psychiatric institutions is usually low. In Germany and Austria, for instance, less than 10% of all convicted sexual offenders are found legally to be dangerous owing to a “severe mental illness” and therefore subsequently detained in forensic psychiatric facilities (1). Although empirical findings support the association between increased offending in general and diagnoses such as schizophrenia or affective illness, particularly when mediated by substance misuse (2), the extant literature has not demonstrated a robust association between a major mental illness per se and sexual offending (3). The existing literature has demonstrated sexual violence to be associated with learning disabilities, substance abuse, personality disorders, and sexual disorders (4, 5). Indeed, research has demonstrated that up to 90% of incarcerated sexual offenders have at least one psychiatric disorder (for more details see 6). In Austria and Germany, being psychiatrically detained requires the offender to be diagnosed with a “severe mental disorder” independent from his or her criminal responsibility (7). Most critically, the disorder must be associated with risk of future reoffending when psychiatric placement is questioned (8, 9).

Ultimately, in constitutional states, decisions about a psychiatric detainment are court decisions. They heavily rely on expert witnesses, who have to certify whether: 1) the offender has a psychiatric diagnosis, 2) the diagnosis is causally linked to the offense for which s/he is accused, 3) if the diagnosis meets the criteria of a severe disorder (i.e., impairing or limiting the individual to act freely, responsibly, and with moral self-determination), and 4) whether the disorder renders the individual to be a high risk for reoffending, absent effective treatment (10). Since detainment in psychiatric hospitals resulting from a finding of dangerousness is indeterminate, and release is contingent on the reduction of risk, valid appraisals of risk and its reduction are of utmost importance. Risk in sexual offenders can effectively be captured by several well validated risk assessment instruments (11); however, some of those instruments, such as the Static-99 and its revision, the Static-99R, are comprised only of static items (e.g., criminal history, offender and victim demographics; 12). Although such instruments typically reliably predict sexual recidivism and inform risk classification (13), their use with mentally ill offenders is limited given that their risk for sexual violence must be reduced to form a compelling argument for release.

Risk relevant change cannot be communicated by means of categorial diagnoses, particularly if the diagnoses connote some form of long-term impairment or vulnerability for a distinct pattern of behavior, such is the case with substance use disorders, paraphilias, or personality disorders. There has yet to be an empirically supported model to translate clinical change into decreased sexual recidivism among psychiatrically detained sexual offenders. This is a significant limitation of psycholegal practice in several respects. First, offenders and treatment service providers face uncertainty as to which specific interventions contribute to the ultimate goal of risk reduction. Second, even if treatment occurs, offender clientele may have benefited minimally (i.e., in terms of risk reduction) or they may even deteriorate owing to unwanted adverse effects of psychotherapy and institutionalization (e.g., dependence from the therapist, stigmatization, suicidal ideation), and continue to pose a substantial recidivism risk upon release. Third, even when these individuals make meaningful strides in treatment, they may still nonetheless be assessed as sexually dangerous or acutely mentally ill, resulting in unnecessary detention in high secure facilities (14).

The Violence Risk Scale–Sexual Offense version (VRS-SO; 15) is a risk assessment and treatment-planning tool that identifies criminogenic needs to be targeted for sexual offense specific treatment. In addition, the VRS-SO assesses change in risk relevant targets and, through use of logistic regression algorithms, can translate such changes into an adjustment of reoffense probabilities (16). Dynamic item ratings further aid understanding of the individual case and inform case formulation. They can be arranged into three factors—sexual deviance, criminality, and treatment responsivity—corresponding to established relevant risk-need domains in sexual offenders (5). Psychometric research has demonstrated that VRS-SO total scores, its three factor domains, and dynamic change scores can predict sexual and violent recidivism for sexual offenders released directly from prison or treated in institutional programs (17).

In the current study, we investigated the predictive properties of DSM-IV-TR diagnoses (18) in a sample of psychiatrically detained and subsequently released sexual offenders. All individuals—prior to release—had been assessed for future sexual violence risk by expert witnesses and had been appraised to be low risk for reoffending. Nevertheless, 12% reoffended sexually, and 21% were psychiatrically redetained or returned to custody for new crimes within a 7-year period. The VRS-SO was used to assess dynamic risk factors at time of admission to the facility and at time of release. Accordingly, we examined associations between VRS-SO scores and changes in reoffending after a minimum of three years follow-up after discharge. Our primary foci were the predictive properties of psychiatric diagnoses contrasted with that of dynamic risk factors and changes therein. We hypothesized that categorical diagnoses would be less useful and informative in appraising future risk than would the VRS-SO and the changes monitored in its dynamic factors.

## Materials and Methods

### Sample

The present study sample comprised all adult male sexual offenders (*N* = 91) in Austria previously placed into a psychiatric facility as a result of a criminal court decision and released between 2008 and 2012. All offenders had been declared to no longer meet the legal criteria for dangerousness by a criminal court as a prerequisite for release. In their index offenses, *n* = 38 (41.8%) men had sexually assaulted adults and *n* = 53 (58.2%) had sexually offended against minors (i.e., below age 14).

Two of the men died during their hospitalization and 14 died during the follow-up period; however, for those who died postrelease, there was sufficient follow-up time to be included in the study sample (*M* = 3.05 years, *SD* = 2.19). High mortality rates in repeated violent offenders have been reported elsewhere (19). The mean age at release (or death in custody) was 53.32 years (*SD* = 14.04) and the sample was followed up an average 7.17 years (*SD* = 2.47) in the community. Reoffense data were available for *n* = 85 offenders, of whom *n* = 11 (12.1%) reoffended sexually (all contact offenses) while *n* = 19 (20.9%) were returned to prison or psychiatrically rehospitalized for a new criminal offense. Reliable diagnostic data from assessment at the time of intake and release was available for *n* = 74 offenders. Sufficient information from file was available to complete VRS-SO pretreatment ratings for *n* = 70 cases and posttreatment ratings for *n* = 57 men.

It is the inherent obligation of the Federal Evaluation Centre for Violent and Sexual Offenders (FECVSO) to continuously evaluate the accuracy of its risk assessment approaches. The FECVSO is a subdivision of the Austrian Ministry of Justice. This evaluation study, therefore, was performed in line with the legal and ethical standards of the Austrian Ministry of Justice and the National and European Data Protection Act. It also relates to the Recommendations of the Committee of Ministers to Member States (Counsel of Europe) Concerning Dangerous Offenders (20) and the Directives of the European Parliament and of the Council on Combating the Sexual Abuse and Sexual Exploitation of Children (21). The study was approved on ethical grounds by the Permanent Control Board for Psychiatrically Detained Patients of the Austrian Ministry of Justice. All high ethical and legal standards concerning research on a vulnerable population have been adhered to accordingly.

### Measures

**VRS-SO.** The VRS-SO comprises seven static items (e.g., criminal history, offender, and victim demographics) and 17 dynamic (i.e., potentially “changeable”) items reflecting domains of psychological, social, emotional and interpersonal functioning. All items are empirically, theoretically, or conceptually linked to risk for sexual reoffending. Items are rated on a 4-point (0, 1, 2, 3) ordinal scale; items receiving a 2 or 3 rating are considered criminogenic and prioritized for treatment. Change is captured by a modified version of the transtheoretical model of change which outlines the cognitive, behavioral, and experiential dynamics as the individual attempts to change identified areas of concern (22). Five stages are defined for each dynamic item: precontemplation (no insight or unwillingness to change); contemplation (awareness of problem area and motivation to change); preparation (preliminary use of skills and strategies, although lapses may be frequent); action (sustained use of skills and strategies, with lapses infrequent); and maintenance (generalization and transfer of skills over an extended period of time and across a range of contexts). Medical and psychological reports about the presence or absence of criminogenic needs were reviewed, as were files documenting treatment progress. Moreover, observed and documented behaviors within the clinic corresponding to offense analogue behaviors (OABs; i.e., offense linked proxy behaviors) and offense replacement behaviors (ORBs; i.e., prosocial skills and strategies) were monitored. A particular emphasis was placed on the offender´s transition from “talk” to “walk,” meaning a credible and transparent change of behavior actively demonstrating this will and his efforts to live in the community sexual offense-free.

Items with a rating of 2 or 3 are assigned a baseline stage at time of placement and the stage is rerated at time of release. Progression from one stage to the next, in the direction of improvement, is credited with a 0.5-point reduction, two stages, 1.0 points, and so on. All ratings were completed retrospectively by S.H. and A.B. blind to recidivism outcome. Both are experienced clinical forensic psychologists and had been trained extensively in the use of the VRS-SO. Interrater reliability of the VRS-SO from prior research on an Austrian prison sample demonstrated good to excellent reliability (*ICCs* = .93 to.98; 23).

**Static-99.** Static-99 and its revision, the Static-99R, are the best validated risk assessment instruments for adult male sexual offenders. Both are 10-item static actuarial sexual violence risk assessment measures used to assess sexual offense risk (24). Meta-analyses robustly support the predictive accuracy of Static-99R for sexual recidivism (AUC = .72); however, since the Static-99 outperforms the Static-99R in German speaking offender samples, we used the Static-99 in our analyses (25). The Static-99 was coded retrospectively by trained forensic psychologists (S.H., A.B.). Interrater reliability has been demonstrated to be excellent (26). Ratings of the Static-99 were made independently from, and blind to, psychiatric diagnoses or recidivism outcome.

### Diagnoses

Diagnoses assigned by expert witnesses during trial and release procedure were recoded and translated into Axis I and for Axis II disorders according to diagnostic criteria of DSM-IV-TR (18) by the same psychologists who rated the VRS-SO and a psychiatrist (R.E.). Diagnoses did not change substantially between the timepoints; however, if diagnoses were different between admission and release, the more credible diagnosis was employed for this study. Paraphilic disorders were coded in the same way according to the diagnostic criteria of the DSM-IV-TR (6).

### Recidivism Variables

Recidivism data was obtained from the Austrian Ministry of Internal Affairs. Sexual recidivism was defined as any new conviction for any sexually motivated contact or noncontact offense. Reimprisonment was defined as any reoffense leading to a subsequent incarceration or psychiatric placement. *N* = 69 offenders were available for follow-up.

### Statistical Analyses

Based on previous research on the prevalence of mental disorders in offender populations (6), single diagnoses were combined to the following diagnostic categories: psychotic disorders, mood disorders, anxiety disorders, eating disorders, impulse control disorders, personality disorders (Cluster A, B, C), and paraphilias. Owing to their putative forensic relevance, we listed all Cluster B disorders separately. Differences in prevalence rates between offenders who relapsed and those who succeeded were analyzed using χ^2^-tests. A putative relationship between continuous data—such as VRS-SO scores and the Static-99—and recidivism was examined through area under the receiver operating curve (AUC) statistics. Values of .56, .64, and .71 represent small, medium, and large effects, respectively (27). We also computed residualized change scores for the three factors through regressing the change score on a given pretreatment score and obtaining the residual; AUCs were computed on the residuals to examine to what extent changes on the VRS-SO factors were associated with decreased recidivism, controlling for pretreatment score. For change outcome analyses, the AUCs were computed with the direction of the binary recidivism criterion variable reversed, such that positive AUCs for change scores would represent inverse associations with outcome, and their magnitudes could be interpreted per the Rice and Harris (27) guidelines.

## Results

Diagnoses of *n* = 74 sexual offenders were available for analysis (see **Table 1**). Of those, *n* = 47 (63.3%) were diagnosed with a DSM-IV-TR Axis I mental disorder, *n* = 69 (93.3%) received an Axis II disorder diagnosis, and *n* = 56 (75.7%) had a paraphilic disorder. None of the psychiatric diagnoses significantly predicted sexual recidivism or reimprisonment, except for exhibitionistic disorder (both outcomes) and a history of substance abuse (any new conviction leading to imprisonment).

**Table 1:** Axis I, Axis II and paraphilic disorders in the total group, in reoffenders and non-reoffenders.

		Sexual reoffense					Reimprisonment			
	Sexual offenders (*n* = 74)	Non-reoffenders (*n* = 60)	Reoffenders (*n* = 9)	F/χ^2^	*p*		Non-reoffenders (*n* = 53)	Reoffenders (*n* = 16)	F/χ^2^	*p*
Age	45.94 (*SD* = 13.32)	45.98 (*SD* = 13.47)	45.37 (*SD* = 13.01)	.00	.958		46.34 (*SD* = 13.11)	44.62 (*SD* = 13.37)	.20	.654
Static-99	4.66 (*SD* = 2.43)	4.47 (*SD* = 2.40)	6.0 (*SD* = 2.49)	2.86	.096		4.38 (*SD* = 2.45)	5.60 (*SD* = 2.17)	3.00	.088
VRS-SO pretreatment total	46.94 (*SD* = 9.36)	46.09 (*SD* = 9.35)	52.61 (*SD* = 7.53)	3.97	**.050**		45.73 (*SD* = 9.28)	50.97 (*SD* = 8.71)	4.04	**.049**
*Any Axis I disorder*	*47 (63.5%)*	*37 (66.7%)*	*6 (61.7%)*	*.08*	*.773*		*31 (58,5%)*	*12 (75%)*	*1.43*	*.232*
Mood disorders	7 (9.5%)	4 (6.7%)	2 (22.2%)	2.39	.122		4 (7.5%)	2 (12.5%)	.38	.538
Anxiety disorders	3 (4.1%)	3 (5%)	0	.47	.493		3 (5.7%)	0	.95	.331
Psychotic disorders	4 (5.4%)	3 (5%)	0	.47	.493		3 (5,7%)	0	.95	.331
Eating disorders	0	0	0				0	0		
Impulse control disorders	0	0	0				0	0		
Substance misuse – drugs	8 (10.8%)	5 (8.3%)	1 (11.1%)	.80	.783		4 (7.5%)	2 (12.5%)	.38	.538
Substance dpendence – drugs	5 (6.8%)	5 (8.3%)	0	.01	.369		4 (7.5%)	1 (6.3%)	.03	.861
Substance misuse – alcohol	22 (29.7%)	15 (25.0%)	5 (55.6%)	3.55	.060		12 (22.6%)	8 (50%)	4.47	**.035**
Substance dependence – alcohol	17 (23%)	14 (23.3%)	1 (11.1%)	.69	.410		11 (20.8%)	4 (25%)	.13	.718
*Any Axis II disorder*	*69 (93.2%)*	*56 (93.3%)*	*8 (88.9%)*	*.23*	*.632*		*49 (92.5%)*	*15 (93.8%)*	*.03*	*.861*
Cluster A PD	10 (13.5%)	7 (11.7%)	3 (33.3%)	2.97	.085		7 (13.2%)	3 (18.8%)	.31	.581
Cluster B PD	56 (75.7%)	44 (73.3%)	7 (77.8%)	.07	.777		38 (71.7%)	13 (81.3%)	.58	.446
Histrionic PD	9 (12.2%)	7 (11.7%)	0	1.17	.280		6 (11.3%)	1 (6.3%)0	.35	.556
Narcissistic PD	23 (31.1%)	19 (31.7%	3 (33.3%)	.01	.920		17 (32.1%	5 (31.3%)	.00	.950
Borderline PD	33 (44.6%)	25 (41.7%)	5 (55.6%)	.61	.433		20 (37.7%)	10 (62.5%)	3.01	.080
Antisocial PD	35 (47.3%)	28 (46.7%)	4 (44.4%)	.02	.901		22 (41.5%)	10 (62.5%)	2.18	.140
Cluster C PD	7 (9,5%)	5 (8.3%)	2 (22.2%)	1.66	.198		5 (9.4%)	2 (12.5%)	.13	.722
PD NOS	8 (10.8%)	8 (13.3%)	0	1.36	.244		7 (13.2%)	1 (6.3%)	.58	.446
*Any Paraphilic disorder*	*56 (75.7%)*	*44 (73.3%)*	*9 (100%)*	*3.13*	*.077*		*41 (77.4%)*	*12 (75%)*	*.04*	*.845*
Exhibitionistic disorder	11 (14.9%)	6 (10%)	4 (44.4%)	7.48	**.006**		4 (5.5%)	6 (37.5%)	8.99	**.003**
Fetishitic disorder	1 (2.7%)	2 (3.3%)	0	.31	.578		2 (3.8%)	0	.62	.430
Frotteuristic disorder	3 (4.1%)	3 (5%)	0	.47	.493		3 (5.7%)	0	.95	.331
Pedophilic disorder	38 (51.4%)	28 (46.7%)	7 (77.8%)	3.03	.082		27 (50.9%)	8 (50%)	.00	.947
Exclusive pedophilic disorder	13 (17.6%)	9 (15%)	3 (33.3%)	1.83	.176		8 (15.1%)	4 (25%)	.84	.360
Sexual masochism	3 (4.1%)	3 (5%)	0	.47	.493		3 (5.7%)	0	.95	.331
Sexual sadism	13 (17.6%)	12 (20%)	1 (11.1%)	.40	.505		11 (20.8%)	2 (12.5%)	.55	.459
Voyeuristic disorder	11 (14.9%)	8 (13.3%)	2 (22.2%)	.50	.480		6 (11.3%)	4 (25%)	1.86	.173
Paraphilia NOS	4 (5.5%)	3 (5%)	1 (11.1%)	.54	.464		3 (5.7%)	1 (6.3%)	.00	.930

Significant p-values in bold font.

The Static-99 total score for the released sample was *M* = 4.66 (SD = 2.43) representing the risk category referenced as Level IVa or above average (6, 13). The total score of the VRS-SO (pretreatment) was *M* = 46.93 (*SD* = 9.34), which was also Level IVa, above average (16). The VRS-SO pretreatment dynamic score was *M* = 37.33 (*SD* = 6.71). Pretreatment sexual deviance, criminality, and treatment responsivity factor scores were *M* = 11.71 (*SD* = 4.19), *M* = 11.40 (*SD* = 6.71), and *M* = 9.54 (*SD* = 2.22), respectively.

The predictive associations between structured risk assessment scores and outcome are reported in **Table 2**. Static-99 and VRS-SO static factor scores did not significantly predict any of the reoffense categories; however, VRS-SO dynamic factor scores (pretreatment) significantly predicted sexual recidivism and a new prison/hospitalization term at broadly moderate magnitude. VRS-SO posttreatement scores also had moderate in magnitude associations with sexual recidivism and general returns to custody, but in this smaller subsample with posttreatment scores, the AUCs did not attain significance. The VRS-SO total score (i.e., static + dynamic) also significantly predicted sexual recidivism. In terms of the three broad dynamic factors, the VRS-SO Sexual Deviance factor was significantly associated with sexual reoffending when scored at time of placement (*AUC* = .70, *p* = .005), and improved in predictive accuracy after treatment at time of release (*AUC* = .74, *p* < .001), with moderate to high AUC magnitudes. The criminality and treatment responsivity factors were not significantly predictive of sexual recidivism but were predictive of any reimprisonment or new psychiatric placement (see **Table 2**).

**Table 2:** AUC Values for the VRS-SO and the Static-99 Prediction of Sexual Recidivism and Reimprisonment.

Measure	Sexual recidivism				Reimprisonment			
	n	AUC	*p*	95%CI^a^	n	AUC	*p*	95% CI^a^
Static-99	77	.63	.174	.51, .74	77	.61	.107	.49, .72
VRS-SO								
VRS-SO pretreatment total	70	.71	**.014**	.59, .80	70	.65	.057	.52, .76
VRS-SO static	70	.65	.090	.53, .70	70	.59	.294	.47, .71
VRS-SO pretreatment dynamic	70	.72	**.007**	.60, .81	70	.70	**.007**	.58, .80
VRS-SO posttreatment dynamic	57	.67	.108	.49, .85	57	.66	.066	.51, .81
Sexual Deviance pretreatment	70	.70	**.005**	.58, .80	70	.54	.600	.42, .66
Sexual Deviance posttreatment	57	.74	**<.001**	.23, .60	57	.51	.887	.50, .74
Residualized change score Sexual Deviance^b^	57	.76	**.014**	.63, .89	57	.61	.228	.46, .75
Criminality pretreatment	70	.51	.941	.39, .63	70	.68	**.029**	.56, .79
Criminality posttreatment	57	.51	.907	.38, .65	57	.63	.119	.49, .76
Residualized change score Criminality^b^	57	.45	.640	.27, .63	57	.44	.520	.27, .62
Treatment Responsivity pretreatment	70	.51	.959	.38, .63	70	.63	.083	.50, .74
Treatment Responsivity posttreatment	57	.56	.386	.44, .71	57	.69	**.007**	.56, 81
Residualized change Treatment Responsivity^b^	57	.58	.484	.40, .75	57	.51	.964	.35, .66

^a^binominal exact. Significant p-values in bold font. Base rate for sexual reoffense 12,1%, base rate for reimprisonment 20,9%. ^b^Positive AUC values for residualized change score associations are interpreted as changes in the factor domains being associated with decreased recidivism.

Finally, residualized change scores for the sexual deviance factor had significant large in magnitude associations with decreased sexual reoffense (*AUC* = .76, *p* = .014); that is, positive changes in this domain in terms of risk reduction were associated with decreased sexual recidivism on release. Specifically, the finding would be interpreted as a 76% probability that a randomly selected sexual nonrecidivist to have made greater risk changes in the domain of sexual deviance, than a randomly selected sexual recidivist, controlling for pretreatment score. Positive changes on the remaining two factors were not significantly associated with changes either recidivism outcome. The change findings broadly bode favorably for the efficacy of treatment and are discussed further.

## Discussion

In Austria and Germany, sexual offenders are psychiatrically detained if the crime is particularly serious, causally influenced by a “severe mental illness,” and they are assessed as high risk for recidivism (7). Detainment is indeterminate with risk and dangerousness must be reexamined annually by the criminal court assisted by psychiatric expert witnesses.

In a cohort of psychiatrically detained sexual offenders released between 2008 and 2012, we evaluated the predictive efficacy of psychiatric diagnosis, static and dynamic risk, and change from treatment. All cases had originally been released on the grounds they were assessed to be low on risk upon discharge; nevertheless, over a 7-year follow-up, 12% reoffended sexually and 21% were imprisoned or psychiatrically rehospitalized for a new criminal act. Despite the fact that the sample had a comparably high *a priori* risk level (the Static-99 and VRS-SO scores allocated the sample to an “Above Average” risk category; 16, 28), the relapse rates were still comparatively high given that offenders had been extensively treated and only those with a positive (clinical) prognosis were released.

The types of severe disorders leading to a psychiatric detainment for sexual offenders are not usually major mental illnesses (e.g., mood disorder or psychoses; 3), but instead tend to be personality disorders and paraphilias, representing long-term vulnerabilities (5). Similarly, in our sample, psychotic disorders were only present in 5% of cases, while personality disorders (93%) and paraphilic disorders (76%) were by far most common. These numbers support the high-risk nature of this population with rates of disorders considerably higher than in prison samples (6). Of those diagnoses, only exhibitionistic disorder predicted sexual reoffending while a history of alcohol misuse predicted any new prison term or psychiatric placement. These findings corroborate the marginal associations found elsewhere between categorial diagnoses and reoffense in sexual offenders (4). Furthermore, the criminological variables of the Static-99, usually robustly linked to reoffense, did not significantly predict either recidivism outcome in our sample.

According to the Austrian law, the presence of a psychiatric diagnosis causally linked to the offense is required for psychiatric placement. And yet personality disorders, substance related disorders, and paraphilic disorders are stable in nature, and one may not expect individuals to emerge effectively symptom free of such diagnoses from their course of treatment. Most of psychiatric diagnoses, in our sample, were not meaningfully associated with sexual reoffense rates, the exception being exhibitionistic disorder, which is well documented to have increased risk for reoffending (29).

If not the diagnosis itself, but rather the treatability of the respective diagnosis is a predictive factor for future reoffending, the more important it is to have a clinical tool monitoring the control and management of disorder-related negative behaviors and potential decreases in risk. One such tool is the VRS-SO. The VRS-SO not only captures risk related criminogenic needs, but also risk reducing behavior indicating improvements in those areas of need. In our study, dynamic factors (criminogenic needs) of the VRS-SO could be shown to be predictive for sexual reoffense and for a further imprisonment or psychiatric placement. The sexual deviance factor of the VRS-SO was found to be of particular interest: not only that it was *a priori* strongly predictive of sexual reoffending, but scores in this domain improved in their predictive validity for this outcome when change information was included; change itself was significantly related to non-reoffending. The results reinforce the efficacy of sexual offense specific treatment to address problems in sexual self-regulation, as measured by this factor, given that positive changes measured pre and posttreatment, were significantly associated with decreased sexual recidivism.

By contrast, the criminality and treatment responsivity factors were found to be significant predictors of general recidivism only, although change was not significantly related to outcome. In all, these findings corroborate those found by Beggs and Grace (17), who demonstrated in their treated New Zealand sample, that changes in the sexual deviance factor of the VRS-SO had the strongest association of the three factors to the desistance of further sexual offenses.

The most apparent limitations of this study are the relatively small sample size and the retrospective design. These limitations, however, are offset by virtue of the fact that our analyses comprise a sample of psychiatrically placed and released sexual offenders assessed for risk with the VRS-SO at admission and release, and subsequently followed up for 7 years postrelease. In addition, this is the first study empirically contrasting treatment outcome with reoffense data of psychiatrically detained and released sexual offenders. Finally, our data support the utility of the VRS-SO at least for psychiatrically detained sexual offenders in Austria and Germany, when risk relevant change must be captured and communicated. Studies with larger samples and in a prospective research design are needed to empirically strengthen the utility of the VRS-SO for this offender population.

## Data Availability Statement

The datasets generated for this study will not be made publicly available. Data requests have to be individually authorized by the Federal Ministry of Justice, Austria.

## Ethics Statement

The study “The predictive properties of psychiatric diagnoses, dynamic risk and dynamic risk change assessed by the VRS-SO in forensically admitted and released sexual offenders” is a study conducted solely through retrospective data analysis. It was ordered and approved by the Permanent Control Board for Psychiatrically Detained Patients (Ständiger Controllingbeirat Maßnahmenvollzug) of the Austrian Ministry of Justice. All high ethical and legal standards concerning research on a vulnerable population are guaranteed.

## Author Contributions

RE designed the research study and supervised SH, AB, and SD. He did all statistical analyses and wrote up the paper. SH and AB completed the VRS-SO ratings and translated diagnoses of the files into DSM-IV-TR diagnoses. SD analyzed and computed reoffense data. DT reviewed and revised an earlier draft of the manuscript. He also contributed to the analyses of the psychiatric diagnoses. MO provided consultation on data analysis and interpretation, reviewed and revised an earlier draft of the manuscript, including contributing new content to the *Materials and Method* *s* and *Results*, and approved the submitted version.

## Conflict of Interest

The authors declare that the research was conducted in the absence of any commercial or financial relationships that could be construed as a potential conflict of interest.
